# Assessing the Analgesic Efficacy of Lumbosacral Epidural Morphine in Cats Undergoing Ovariohysterectomy: A Comparative Study of Two Doses

**DOI:** 10.3390/vetsci11080360

**Published:** 2024-08-09

**Authors:** Ludimilla C. T. Martins, Jéssica B. Guimarães, Henrique T. Ferraz, Flávia Augusta de Oliveira, Leidiane de S. Gomes, Clóvis Júnior C. Chafes, Thalita de C. C. Santos, Kaline Ogliari, Reiner S. de Moraes, Diego Ribeiro, Dirceu Guilherme de Souza Ramos, Thiago André S. de S. Rocha, Doughlas Regalin

**Affiliations:** 1 School of Veterinary Medicine and Animal Bioscience, Federal University of Jataí (UFJ), Jataí 75804-068, Brazil; ludimillacristina@discente.ufj.edu.br (L.C.T.M.); jessybueno92@gmail.com (J.B.G.); htferraz@ufj.edu.br (H.T.F.); clovischafes@gmail.com (C.J.C.C.); vet.thalitac@gmail.com (T.d.C.C.S.); kalineogliari@hotmail.com.br (K.O.); dguilherme@ufj.edu.br (D.G.d.S.R.); thiago.rocha@ufj.edu.br (T.A.S.d.S.R.); 2 School of Veterinary Medicine, North Tocantins Federal University (UFNT), Araguaína 77818-530, Brazil; fla.anestesio@hotmail.com; 3 Residency in Clinics and Surgery of Companion Animals, Federal University of Goiás (UFG), Goiânia 74690-900, Brazil; leidianeveterinaria@gmail.com; 4 Department of Veterinary Clinics, School of Veterinary Medicine and Animal Science, São Paulo State University (UNESP), Botucatu 18618-681, Brazil; rs.moraes@unesp.br (R.S.d.M.); diego.ribeiro@unesp.br (D.R.)

**Keywords:** analgesia, cats, pain, propofol, rescue

## Abstract

**Simple Summary:**

We evaluated the analgesic and postoperative effects of epidurally administered opioids and local anesthetics in 20 cats that underwent elective ovariohysterectomy (OH). Propofol was used as the general anesthetic. The animals were divided into three groups according to the dose of the epidurally administered morphine. Therefore, it was necessary to use fentanyl to complement analgesia in all groups. Postoperatively, 83%, 28%, and 7% of the animals without morphine administration, with a lower dose of morphine, and with a higher dose of morphine, respectively, required additional analgesia. In conclusion, in cats undergoing OH, epidural morphine at the doses used did not eliminate the need for intraoperative rescue analgesia but did reduce the need for postoperative analgesia.

**Abstract:**

Opioids are administered epidurally (PV) to provide trans- and postoperative analgesia. Twenty healthy female cats aged between 6 and 24 months and weighing between 2 and 3.7 kg, undergoing elective ovariohysterectomy (OVH), were induced with propofol (8 mg/kg), followed by continuous infusion (0.1–0.4 mg/kg/min). Three groups were defined: CG (0.1 mL/kg of iodinated contrast, *n* = 6), G0.1 (0.1 mg/kg of morphine, *n* = 7), and G0.2 (0.2 mg/kg of morphine, *n* = 7) per VP. All received 0.1 mL/kg of iodinated contrast per VP and injection water to obtain a total of 0.3 mL/kg. Heart rate (HR), systolic blood pressure (SBP), temperature, expired CO_2_, oxygen saturation, and number of rescue analgesics were monitored. Postoperatively, a multidimensional scale was used to assess acute pain in cats for 12 h. The mean HR and SBP in the CG were higher at the time of maximum noxious stimulation and required fentanyl in all groups. Postoperatively, 83%, 28%, and 7% of the animals in CG, G0.1, and G0.2, respectively, received rescue analgesia. In cats undergoing OVH, epidural morphine at doses of 0.1 and 0.2 mg/kg did not prevent the need for intraoperative rescue analgesia but reduced the postoperative analgesic needed.

## 1. Introduction

The growing population of domestic cats in recent years has raised concerns regarding the well-being of these animals, which frequently require specific care. Consequently, pain assessment scales have been validated for the evaluation and identification of acute pain in feline species. To achieve this goal, studies involving different drugs and doses are essential to ensure the accuracy and effectiveness of pain treatments [1].

The treatment of acute pain involves the use of opioids, nonsteroidal anti-inflammatory drugs, and local anesthetics. Opioids and local anesthetics, either alone or in combination, are commonly administered epidurally. They provide analgesia during both the intra- and postoperative periods and are considered part of a multimodal analgesic protocol [2,3,4]. Although there has recently been the possibility of sacrococcygeal or intercoccygeal administration of neuroaxis blocks in domestic cats, it is important to understand that the lumbosacral approach to neuraxial anesthesia in cats can result in dural sac puncture, as the spinal cord terminates at S2 in domestic cats. Therefore, the intercoccygeal approach is preferred. However, because it is a widely described and used technique, lumbosacral puncture can also be performed with great caution by trained professionals [4].

Morphine is an opioid frequently administered via the intramuscular, subcutaneous, intrathecal, intra-articular, epidural, or intravenous routes [5]. It exhibits high affinity for μ receptors and low affinity for κ and δ receptors, in addition to having low liposolubility, resulting in slow penetration of the blood–brain barrier and consequently prolonging the drug’s latency [6,7]. Opioids administered via the epidural or intrathecal route are commonly combined with local anesthetics. However, when there is a need for greater cranial distribution of blocks or for primary use in postoperative analgesia, opioids can be used alone in different spinal segments [8,9].

A study by DeRossi et al. [10] evaluated postoperative pain control in cats undergoing ovariohysterectomy (OVH) via epidural administration of lidocaine (4 mg/kg) alone or in combination with morphine (0.1 mg/kg) or methadone (0.3 mg/kg). A longer time to rescue analgesia was demonstrated in protocols that used opioids. However, there are few studies on domestic cats revealing the efficacy of different doses of morphine and its use as a sole agent. Considering the anesthetist’s familiarity with the lumbosacral approach and the aim of visualizing the contrast agent administered, the present study aimed to evaluate intra- and postoperative analgesia using two different doses of morphine administered solely via the lumbosacral route in cats undergoing elective OVH.

## 2. Materials and Methods

### 2.1. Ethical Approval

This study was approved by the Animal Ethics Committee (CEUA) of the Federal University of Jataí (UFJ) under protocol number 14/2021.

### 2.2. Animals

Twenty female cats (*Felis catus*) sourced from the routine activities of the Veterinary Hospital at UFJ were included in this study, with owner approval obtained through the Anesthetic and Surgical Procedure Authorization Form and the Owner’s Informed Consent (Supplementary Material File S1).

One week before the surgical procedure, anamnesis and physical examination were performed. Blood was collected from the jugular vein for complete blood count (CBC) and hepatic and renal biochemistry analysis. Additionally, ultrasonography was performed to rule out pregnancy or estrus and thoracic radiography was used to exclude abnormalities that could interfere with anesthesia or surgery. The patients were then referred to the Anesthesiology and Surgery Department for elective OVH.

### 2.3. Anesthesia Procedure

The day before the surgical procedure, hair was removed from the right and left thoracic limbs for cannulation of the cephalic vein, and the palmar region of the metacarpus was prepared for systolic blood pressure assessment. The Doppler probe was positioned between the carpus and metacarpal pads and held under light pressure by an evaluator to ensure good contact. The position of the probe was carefully adjusted until pulsatile blood flow in the digital artery was detected. The cuff width used was 30–40% of the forelimb circumference. Newly developed manometers were used in this study. The animals were allocated into three groups: the control group (CG, *n* = 6), which received 0.1 mL/kg iodine in combination with water to obtain a final volume of 0.3 mL/kg, and the G0.1 (*n* = 7) and G0.2 (*n* = 7) morphine groups, which received 0.1 mg/kg and 0.2 mg/kg epidural morphine, respectively. In both groups, 0.1 mL/kg of iodine contrast agent and water were injected, to obtain a 0.3 mL/kg solution.

In this study, designations such as M for intraoperative and T for postoperative measurements were used to avoid confusion. Two animals were excluded from the study because contrast marking indicated that the injection was outside the puncture site. Additionally, one animal in the CG experienced excessive pain (immediate postoperative allodynia) and did not complete the evaluation period.

Prior to surgery, each animal was placed in a radiography room, where the cephalic vein was accessed using a 22 G (25 mm) catheter. Anesthesia was induced with an initial dose of 8 mg/kg propofol, and 0.2 mL of lidocaine was instilled into the epiglottis before intubation using a Murphy-type tube appropriate for the size of the animal. All cats were maintained on spontaneous ventilation in a system without gas rebreathing (baraka), with the aid of an oxygen concentrator at a flow rate of 3 L/min and an FiO_2_ of 95%. After induction, the epidural and abdominal hair were removed. The temperature of the surgery room was set to 24 °C.

All groups were maintained with continuous propofol infusion at a variable rate, adjusted between 0.1 and 0.4 mg/kg/min in a time-dependent manner [11], starting with 0.4 mg/kg/min at M0. The cats were positioned in lateral recumbency and left without stimuli for 10 min to standardize the anesthetic plan. Post 10 min, baseline parameters (M0) were recorded for the animals under spontaneous ventilation after intubation, followed by a pre-epidural radiograph obtained by the technician.

The animal was then positioned in sternal recumbency, with the hind limbs cranially retracted, and the lumbosacral region aseptically prepared for the placement of a fenestrated surgical drape. Through palpation, the space between the seventh lumbar vertebra (L7) and the first sacral vertebra (S1) was identified, and a 22 G Tuohy needle (50 × 7 mm) was inserted through the skin into this space. Once the needle penetrated the skin, it was advanced into the L7-S1 intervertebral space until the ligamentum flavum was punctured into the epidural space. In the absence of blood or cerebrospinal fluid, a syringe was attached, and epidural administration was performed. This technique was performed as described by Grubb and Lobprise [4].

To ensure that epidural administration was performed in the correct space, the absence of spontaneous cerebrospinal fluid (CSF) reflux and the lack of fluid return under aspiration with a glass syringe were verified. Thus, we confirmed that it was not injected in the subarachnoid space. Animals without contrast demarcation in the epidural space on radiography were excluded.

**Figure 1:**
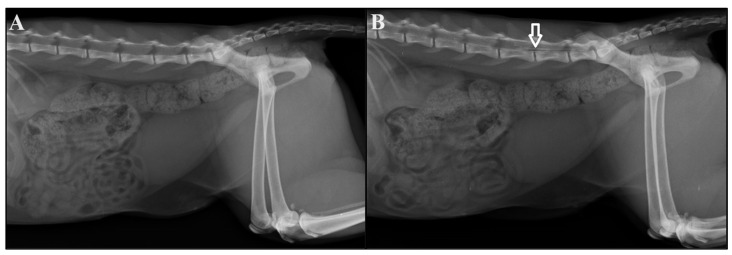
Radiography images before (**A**) and after (**B**) the administration of 0.1 mg/kg (G0.1) or 0.2 mg/kg (G0.2) morphine and iohexol contrast agent in the epidural space in cats anesthetized with a continuous infusion of variable-dose propofol and undergoing ovariohysterectomy. The white arrow in (**B**) indicates the extent of contrast with the local anesthetic.

After epidural administration, a second radiograph was obtained to confirm injection into the lumbosacral space (Figure 1) as described by Ramos et al. [12]. The animal was then transferred to the surgical center on a stretcher, placed on the operating table in dorsal recumbency, and connected to a mechanical ventilator with the following settings: cyclical pressure ventilation mode, PInsp 10 cm/H_2_O, respiratory rate (RR) of 12 bpm (breath per minute), I:E ratio 1:2, PEEP 0, and FiO_2_ 100%. Antiseptic measures, surgical drape placement, and surgical table organization were performed over a 30 min period.

After 30 min, a new assessment of baseline parameters (M1) was performed, and the propofol dose was reduced to 0.3 mg/kg/min. During the intraoperative period, new parameter readings were obtained at the following time points: skin incision (M2), clamping of the right ovarian pedicle (M3), clamping of the left ovarian pedicle (M4), clamping of the cervix (M5), celiorrhaphy (M6), and dermorraphy (M7). Approximately 10 and 20 min post initiation of surgery, the propofol dose was reduced to 0.2 mg/kg/min and 0.1 mg/kg/min, respectively. 

If a positive response to surgical stimulation occurred (a 20% increase in heart rate (HR) or systolic blood pressure (SBP) compared to baseline-M0), a fentanyl bolus of 2.5 μg/kg was administered intravenously. In cases of bradycardia (<100 bpm) combined with hypotension (<90 mmHg), the use of intravenous atropine (0.03 mg/kg) was considered.

### 2.4. Trans-Anesthetic Monitoring

During the intraoperative period, HR in beats per min (bpm) through electrocardiography (ECG), end-tidal carbon dioxide (EtCO_2_) in mmHg through capnography, oxygen hemoglobin saturation (SpO_2_) with a pulse oximeter in a multiparametric monitor, rectal temperature (T°C) using a clinical thermometer, and systolic blood pressure (SBP) in millimeters of mercury (mmHg) were evaluated noninvasively using a portable vascular Doppler.

### 2.5. Postoperative Pain Assessment

Postoperative pain was assessed 2, 4, 6, 8, and 12 h post-surgery. Assessments were conducted by two “blinded” evaluators using the UNESP-Botucatu multidimensional pain scale for domestic cats. Animals with a score equal to or greater than eight points on this scale received rescue analgesia involving intramuscular administration of 0.2 mg/kg morphine. Pain was classified based on the following scores: 0–8 (mild pain), 9–21 (moderate pain), and 22–30 (severe pain) [13].

### 2.6. Statistical Analysis

Statistical analysis included the Shapiro–Wilk normality test. Parametric data were subjected to one-way ANOVA, followed by the Student–Newman–Keuls test for between-group comparisons. For within-group comparisons at different time points, one-way repeated measures analysis of variance ANOVA (RM) was used, followed by the Bonferroni correction. Nonparametric data were analyzed using one-way ANOVA (RM) followed by the Friedman test for within-group comparisons and one-way ANOVA followed by the Kruskal–Wallis test for between-group comparisons. Survival analysis and Kaplan–Meier curve analysis were performed to assess the need of rescue analgesia using GraphPad Prism 5.0^®^ software and the log-rank test.

The power analysis of the test was 0.08 and the alpha error (type I) was set at 5%. Statistical significance was considered when *p* < 0.05. 

## 3. Results

The mean body weights of the cats in GC, G0.1, and G0.2 were 2.7 ± 0.5 kg, 2.9 ± 0.5 kg, and 2.6 ± 0.5 kg, respectively, with no difference between groups. All cats were of mixed breed and were aged 6–24 months.

For variables assessed intraoperatively, an increase in HR at M3 compared to that at M0 was observed in CG. In G0.1 and G0.2, a reduction in HR at M1, M2, M6, and M7 compared to that at M0 was observed. The mean HR in CG was greater than that in G0.1 at M3 (Table 1). SBP increased in CG at M3, M4, and M5; in G0.1 at M3, M4, M5, and M6; and in G0.2 at M3 and M4. SBP was greater in CG than in G0.1 and G0.2 at M2, and in G0.2 at M3 (Table 1). Atropine resulted in a 64% increase in HR and a 66% increase in SBP in three of seven cats in G0.1, and two of seven cats in G0.2, all of which were administered before M1.

A decrease in T°C was observed at all time points in CG, G0.1, and G0.2 compared to M0. Moreover, between groups, the T°C values in CG and G0.2 were greater than those in G0.1 at M2 to M7 and M4 to M7, respectively (Table 1).

No significant differences were observed in intraoperative fentanyl rescue events (*p* = 0.87). Additionally, compared with G0.1 and G0.2, CG received more rescue analgesia. More than one rescue was required at M3, M4, and M5 in CG, G0.1, and G0.2 (Table 2).

In the postoperative period, a significant difference in the number receiving rescue analgesia performed with morphine between CG and G0.1 and G0.2 (*p* = 0.0054) was observed. However, no difference between G0.1 and G0.2 was found. With respect to the animals, 83%, 28%, and 7% in CG, G0.1, and G0.2, respectively, received rescue analgesia within 12 h period (Table 3). The pain score assessments are presented in Table 4.

## 4. Discussion

Compared with other species, the production of the active morphine metabolite morphine-6-glucuronide is limited in cats. This metabolite is responsible for some of the observed analgesic effects, and the lack of production of this metabolite in cats may be why morphine (0.1 mg/kg) appears to be less effective than buprenorphine (0.01 mg/kg) in cats undergoing invasive procedures [14]. However, in a study by Stanway et al. [14], administration was performed intramuscularly. The administration of epidural buprenorphine (0.0125 mg/kg) resulted in thermal antinociception for up to 10 h [15] or up to 24 h with a dosage of 0.02 mg/kg [16]. Therefore, morphine doses of 0.1 mg/kg and 0.2 mg/kg were used in our study to identify any differences in analgesic effects between the two doses via the lumbosacral epidural region.

Although discussed by veterinary professionals, epidural anesthesia can be performed using either lumbosacral or sacrococcygeal approaches, with complications being reported as rare in both techniques and no specific complications associated with the choice of the puncture site [4]. In our study, the lumbosacral approach was considered based on professional experience. Therefore, the epidural space assessment technique was performed according to the recommendations of Grubb and Lobprise [4]. Confirmation of the correct execution of the technique is not solely based on the absence of needle resistance. Other methodologies such as ultrasound, neurolocators, contrast administration in the epidural space, and the absence of cerebrospinal fluid in the epidural needle have been employed for verification [17,18,19]. Owing to the unavailability of a neurolocator and the inadequacy of the ultrasound device probe for the size of the animals, the last two techniques were utilized to identify the epidural space and confirm the deposition of the drug and contrast agent, thereby including the animals in our study.

Visceral nociception is the primary stimulus during the intraoperative period, reaching its peak during traction and clamping of the ovarian and uterine pedicles, which is considered the maximum surgical stimulus [20], and could be better identified in CG cats than in G0.1 and G0.2 cats. Anatomically, sensory nerve fibers innervating the canine ovary originate and spread widely between the T10 and L4 medullary segments, with higher concentrations near the thoracolumbar junction (from T13 to L3) [21]. Moreover, postganglionic sympathetic nerves reach the uterus through the hypogastric nerve and adrenergic neurons originating at the uterovaginal junction. Although no studies have reported the origin of the sensory nerve fibers in domestic cats, we suggest that these fibers are anatomically similar to those in female cats [22,23,24]. To confirm the deposition of epidural morphine, all cats in our study underwent radiography before and after contrast and anesthetic administration into the epidural space.

Iohexol, an iodine contrast medium, is used in epidurography and myelography, expanding its use in anesthesiology to identify the presence of local anesthetics and analgesics in the epidural space [25]. In the study by Otero et al. [26], the dose used was 0.2 mL/kg, which is greater than that used in our study. However, the lower dose used in the present study was sufficient to visualize the contrast agent and morphine in the epidural space.

The increase in HR and SBP in the CG compared to that in G0.1 and G0.2 occurred during periods of increased noxious stimuli. This increase, coupled with the high number of rescue interventions, particularly during right ovarian pedicle clamping, was attributed to the lack of a decrease in response to nociceptive stimuli. It is worth noting that morphine exerted its analgesic effect within 30 min of epidural administration, falling within the latency period of 30–60 min, as described by Otero [27], with more pronounced evidence in postoperative rescues. Therefore, during surgery, the use of epidural morphine at doses of 0.1 or 0.2 mg/kg did not provide satisfactory analgesia to abolish the peak moment of nociceptive stimulus during OVH, which is classified as moderate pain. Hence, the significance of multimodal analgesia has become evident through the incorporation of various locoregional analgesic techniques and a range of medications, such as local anesthetics, anti-inflammatory drugs, and opioids, aimed at preventing the progression, perception, and recognition of pain [28].

A decrease in HR was also observed in G0.1 and G0.2 compared with M0. In addition to the high-dose propofol infusion, there was no nociceptive stimulus between M0 and M1, deepening the anesthetic plane from which cardiovascular changes were observed, as mentioned by Mendes and Selmi [29] and Pereira et al. [30]. In their studies, propofol infusion doses of 0.2 mg/kg/min and 0.5 mg/kg/min culminated in a reduction in cats’ HR after 30 min of drug infusion.

Despite the QT interval prolongation effect of opioids, morphine promotes a low risk of bradyarrhythmias at clinical doses [31]. Additionally, the administration of epidural morphine and its absorption potentiated parasympathetic tone, leading to mild bradycardia and hypotension in three of the seven cats in G0.1 and two of the seven cats in G0.2 before M1, similar to the findings of Regalin et al. [32]. In our study, atropine at a dose of 0.03 mg/kg was administered intravenously to reverse the aforementioned effects, as indicated by Grubb et al. [33], increasing HR by 64% and SBP by 66% in G0.1 and G0.2, respectively. The mechanism of action involves blocking muscarinic receptors, resulting in vagal inhibition and increased HR and, consequently, SBP, with a duration of 30 min [34,35]. However, at clinical doses, as in the cats used in our study, it had an antagonistic effect on the parasympathetic nervous system (PNS), with the sympathetic nervous system (SNS) not being activated and maintaining HR and SBP values close to the reference interval. Thus, no significant variations were observed during anesthetic or surgical procedures.

Mechanical ventilation is essential for maintaining eucapnia in healthy animals and those with respiratory diseases. In our study, cats were placed on pressure-controlled mechanical ventilation starting from M1 (the beginning of the surgical procedure). The differences observed under spontaneous ventilation were related to an increase in the RR to maintain a minute volume owing to the minute ventilation reduction caused by propofol. However, when controlled ventilation is applied, as in our study, this suppression is alleviated by increasing the minute volume, resulting in eucapnia maintenance [36,37].

Moreover, a reduction in T°C compared to M0 at all time points was observed. Despite receiving all active and available warming methods, cats still experienced hypothermia, which is a common finding in abdominal surgery [38].

Patients who experienced a supramaximal nociceptive stimulus corresponding to the ovarian pedicles and cervical traction normally received more rescue analgesia. However, no statistically significant increase in the number of rescues during the intraoperative period was observed among GC, G0.1, and G0.2. The cats in the CG did not receive any epidural analgesics, highlighting the analgesic effect of morphine via the lumbosacral route and revealing the presence of receptors located along the dorsal horn of the spinal cord [39]. Similarly, this analgesic effect was confirmed by Dourado et al. [40], who compared intraoperative analgesia in the CG with intramuscular methadone (0.2 mg/kg) and in other groups combined with lidocaine (0.3 mL/kg) and epidural morphine (0.1 mg/kg) in cats undergoing OVH. All control cats required intraoperative fentanyl rescue analgesia, whereas only 40% of the animals administered epidural fentanyl required intraoperative rescue analgesia.

However, morphine administered via the lumbosacral route, whether at a dose of 0.1 mg/kg or 0.2 mg/kg, contributed to postoperative pain control in the cats, reducing the need of rescue analgesia.

The difference between CG and G0.1 and between CG and G0.2 was evident in the rescue analgesia effect of morphine in CG from 2 h to 8 h during the postoperative evaluation period. In both G0.1 and G0.2, morphine exerted an analgesic effect 6 h after surgery, which is consistent with previous studies in which 0.1 mg/kg morphine was administered via the epidural route [9,10,15].

One limitation of our study is that nonsteroidal anti-inflammatory drugs (NSADs) were not included in the protocol and were administered solely after the end of the study, as we aimed to evaluate the effects of epidural morphine. However, for logistical reasons, patients were not on mechanical ventilation at baseline; however, there was no apnea or need for ventilatory supplementation based on the ETCO_2_ values. Another limitation of this study is that IM morphine for postoperative rescue can take 30–60 min to reach its peak effect. Thus, animals with higher pain scores should have been administered IV morphine. Additionally, to determine the sample size in our study, blood pressure was the main parameter for intraoperative rescue, and the average peak blood pressure of patients not using epidural block was considered to be 150 mmHg, while animals receiving the drug via epidural block had pressures between 100 and 120 mmHg. Thus, considering a standard deviation of 30 mmHg, encompassing both normotensive and hypertensive patients, a sample size of 7 animals per group was determined, totaling 14 animals in the epidural groups of the study, with 6 animals in the CG. Thus, despite the statistical tests conducted to calculate the sample size, the study may have been underpowered. Based on regular clinical settings and published studies, a multimodal approach for analgesia is recommended.

## 5. Conclusions

Thus, no detectable difference between the two doses of morphine was observed. Epidural morphine at doses of 0.1 mg/kg and 0.2 mg/kg did not prevent rescue analgesia requirements during the intraoperative period in cats undergoing elective OVH. However, lumbosacral epidural morphine reduced the postoperative analgesic requirements in these animals, suggesting that the doses of morphine administered epidurally in our study were satisfactory for postoperative analgesia in female cats subjected to OVH.

## Figures and Tables

**Table 1:** The mean ± standard deviation values for heart rate (HR), systolic blood pressure (SBP), respiratory rate (RR), end-tidal carbon dioxide (EtCO_2_), oxygen hemoglobin saturation (SpO_2_), and rectal temperature (°C) in the intraoperative period (M0, M1, M2, M3, M4, M5, M6, and M7), baseline, 30 min after baseline, skin incision, clamping of the right ovarian pedicle, clamping of the left ovarian pedicle, clamping of the cervix, celiorrhaphy, and dermorraphy, respectively.

Parameter	Group	M0	M1	M2	M3	M4	M5	M6	M7
HR (bpm)	CG	140 ± 34	126 ± 26	134 ± 24	167 ± 10 ^A^	151 ± 10	152 ± 9	134 ± 31	133 ± 30
	G0.1	154 ± 22	102 ± 14 ^A^	108 ± 22 ^A^	129 ± 15 ^a^	137 ± 11	133 ± 24	121 ± 28 ^A^	118 ± 2 ^A^
	G0.2	154 ± 5	116 ± 24 ^A^	116 ± 22 ^A^	141 ± 24	145 ± 22	140 ± 26	121 ± 20 ^A^	128 ± 2 ^A^
SBP (mmHg)	CG	102 ± 16	95 ± 19	135 ± 19	179 ± 21 ^A^	179 ± 47 ^A^	145 ± 31 ^A^	121 ± 26	122 ± 26
	G0.1	88 ± 15	82 ± 16	82 ± 16 ^a^	134 ± 31 ^A^	135 ± 24 ^A^	133 ± 26 ^A^	128 ± 35 ^A^	114 ± 15
	G0.2	95 ± 23	79 ± 15	86 ± 22 ^a^	128 ± 41 ^Aa^	132 ± 36 ^A^	122 ± 31	99 ± 20	97 ± 23
RR (bpm)	CG	19 ± 5	11 ± 2	12 ± 2	12 ± 2	12 ± 1	11 ± 2	11 ± 1	11 ± 1
	G0.1	16 ± 4	10 ± 2	12 ± 4	11 ± 3	12 ± 3	13 ± 4	13 ± 4	13 ± 4
	G0.2	15 ± 3	11 ± 2	12 ± 1	12 ± 1	12 ± 1	12 ± 1	12 ± 1	11 ± 1
EtCO_2_ (mmHg)	CG	28 ± 6	41 ± 2	40 ± 3	42 ± 2	41 ± 3	40 ± 4	40 ± 4	40 ± 3
	G0.1	28 ± 14	43 ± 13	41 ± 18	40 ± 14	45 ± 10	41 ± 11	43 ± 15	38 ± 2
	G0.2	32 ± 14	43 ± 7	42 ± 7	40 ± 6	38 ± 4	37 ± 4	39 ± 7	38 ± 6
SpO_2_ (%)	CG	95 ± 2	99 ± 1	98 ± 2	99 ± 1	99 ± 1	99 ± 1	99 ± 1	99 ± 1
	G0.1	95 ± 3	98 ± 2	98 ± 2	98 ± 2	99 ± 1	98 ± 2	99 ± 1	98 ± 2
	G0.2	98 ± 3	99 ± 1	99 ± 1	99 ± 1	98 ± 4	99 ± 1	99 ± 1	98 ± 1
Rectal temperature (°C)	CG	38.2 ± 0.5	36.8 ± 1.1 ^A^	36.5 ± 0.8 ^A^	36.4 ± 0.6 ^A^	36.3 ± 00.5 ^A^	36.2 ± 0.7 ^A^	36.0 ± 0.6 ^A^	36.0 ± 0.5 ^A^
	G0.1	38.8 ± 0.3	36.3 ± 1.2 ^A^	35.6 ± 1.4 ^Aa^	35.5 ± 1.0 ^Aa^	35.4 ± 1.1 ^Aa^	35.3 ± 1.2 ^Aa^	34.1 ± 1.1 ^Aa^	34.0 ± 1.4 ^Aa^
	G0.2	38.5 ± 1.2	37.0 ± 1.0 ^A^	36.6 ± 1.1 ^A^	36.4 ± 1.3 ^A^	36.2 ± 1.0 ^Ab^	36.1 ± 1.2 ^Ab^	36.0 ± 1.1 ^Ab^	35.8 ± 1.0 ^Ab^

The uppercase letter “^A^” in the same line indicates a difference from M0 (ANOVA followed by the Bonferroni correction, *p* ≤ 0.05). The lowercase letter “^a^” in the same column indicates a difference from CG, and the lowercase letter “^b^” indicates a difference from G0.1 (one-way ANOVA followed by SNK *p* ≤ 0.05).

**Table 2:** Total number receiving rescue analgesia at different time points with fentanyl administered intravenously at a dose of 2.5 µg/kg intraoperatively.

Moment	CG (*n* = 6)	G0.1 (*n* = 7)	G0.2 (*n* = 7)
M0	0	0	0
M1	0	0	0
M2	1	0	0
M3	11	8	7
M4	12	8	6
M5	9	6	5
M6	3	1	0
M7	3	0	0

The numbers within the table represent the number receiving rescue analgesia.

**Table 3:** Total number receiving rescue analgesia performed postoperatively with 0.2 mg/kg morphine administered intramuscularly (0, 2, 4, 6, 8 and 12 h) post-operation.

Time Points	GC (*n* = 6)	G0.1 (*n* = 7)	G0.2 (*n* = 7)
T0	0	0	0
T2	1	0	0
T4	3	0	0
T6	2	1	0
T8	2	1	1
T12	0	0	0

The numbers within the table represent the amount of rescue analgesia.

**Table 4:** Median (1st and 3rd quartiles) of the total sum obtained through the UNESP-Botucatu multidimensional pain scale in cats anesthetized for OVH with continuous infusion of variable dose propofol, undergoing epidural with injection of sterile water (GC), and morphine at doses of 0.1 mg/kg (G0.1) or 0.2 mg/kg (G0.2).

Group/Time Points	T0	T2	T4	T6	T8	T12
**GC**	0 (0–0)	6 (3–8)	8 (4–15) ^A^	6 (2–8)	4 (1–10)	1 (0–3)
**G0.1**	0 (0–0)	4 (2–5) ^A^	4 (3–4) ^A^	2 (1–7) ^A^	1 (1–4) ^A^	1 (0–1)
**G0.2**	0 (0–0)	4 (1–5) ^A^	2 (1–4) ^A^	1 (1–3) ^A^	2 (0–2)	0 (0–1)

Letter ^A^ in the row indicates difference from M0. Friedman test was performed.

## Data Availability

The datasets generated and/or analyzed in the current study are available from the corresponding author upon reasonable request.

## Supplementary Materials

The following supporting information can be downloaded at: https://www.mdpi.com/article/10.3390/vetsci11080360/s1, File S1:Informed consent form.
