# Supplementary material for: Assessing the Analgesic Efficacy of Lumbosacral Epidural Morphine in Cats Undergoing Ovariohysterectomy: A Comparative Study of Two Doses
Source: Vet Sci. 2024 Aug 9;11(8):360. doi: 10.3390/vetsci11080360 (PMC11358909; doi:10.3390/vetsci11080360)
Supplement: Supplementary file 1 [file vetsci-11-00360-s001.zip › vetsci-3021061-supplementary.pdf]

INFORMED CONSENT FORM

Project title: Analgesic effect of epidural morphine at two different doses in felines undergoing elective ovariohysterectomy (OVH)

Principal researcher's name: Ludimilla Cristina Teles Martins

Corporate name of UFJ and protocol number in CEUA: 14/2021

Study objectives: To investigate the analgesic effects of epidural morphine in the feline species at doses of 0.1 mg/kg and 0.2 mg/kg in healthy female cats undergoing elective ovariohysterectomy (OVH).

Procedures to be performed on the animals: The animals will be received one day (24 hours) before the surgical procedure and will be accommodated in the post-operative room, where they will receive food, water, and will be evaluated for pain level and well-being. Prior to the procedure, they will remain fasting for 12 hours. After anesthetic induction with propofol, epidural anesthesia with morphine (each animal at the pre-established dose randomly assigned) will be administered, followed by maintenance with propofol infusion. The OVH surgical procedure will be performed, and at the end, the animals will be relocated to the post-operative room, where pain analysis will be conducted using the Multidimensional Pain Scale within the first 12 hours after surgery. If the animal experiences pain, analgesic medication (morphine) will be administered. Upon discharge, the patient will receive a prescription for analgesics and anti-inflammatory medications.

Potential risks for the animals: The risks to which the animals participating in the study will be subjected are the anesthetic and surgical risks, as in any other surgical procedure.

Benefits: The animals participating in the study may benefit from the trans and post-operative analgesia provided by the epidural injection of morphine. Overall, the study aims to benefit the scientific community with data and information indicating the best dose, or the dose that provides the best analgesia in female cats undergoing OVH. Thus, other animals

undergoing this procedure in the future will receive epidural anesthesia with the most appropriate dose for the species.

Your authorization for the inclusion of your animal(s) in this study is voluntary. Your animal(s) may be withdrawn from the study at any time without causing any harm to them.

The confidentiality of your personal data will be preserved.

Members of the CEUA or regulatory authorities may request your information, and in this case, it will be directed specifically for the purposes of regular inspections.

The Veterinarian responsible for your animal(s) will be Dr. Ludimilla Cristina Teles Martins**, registered with the CRMV under number** 01598/TO. **Additionally, the principal investigator's team will also be responsible for the well-being of your animal(s) throughout the study and at its conclusion. If necessary, during or after the study period, you may contact the principal investigator or his/her team at the following contacts:**

Emergency phone: (64) 992350489

Team: Doughlas Regalin, Flávia Augusta de Oliveira, Kaline Ogliari, Jéssica Bueno Guimarães, Bruna Ditzel da Costa Regalin, Leuton Scharles Bonfim

Address: Quadra 305 Sul, Rua 6, Qi 19, casa 6B. Setor: Plano Diretor Sul, Palmas-TO.

Phone: (63) 99235-0489

If necessary, during or after the study period, you may contact the Animal Use Ethics Committee/UFG by phone at (62) 3521-1876 or by email at ceua.ufg@gmail.com.

This consent form follows the recommendations of CONCEA Normative Resolution No. 22, dated June 25, 2015.
